# Niagara and Orleans County Veterinary Medical Society

**Published:** 1900-08

**Authors:** Anderson Crowforth

**Affiliations:** Secretary


### SOCIETY PROCEEDINGS.

### NIAGARA AND ORLEANS COUNTY VETERINARY MEDICAL SOCIETY.

This Society convened at the office of the Secretary, Lockport, N. Y., August 11th, at 2 P.M. The President, Dr. W. E. Stocking, called the meeting to order. On roll-call the following officers responded: Drs. W. E. Stocking, Medina, N. Y.; M. D. Williams, Middleport, N. Y.; John Liddle, West Shelby, N. Y.; Wm. Hunter, Niagara Falls, N. Y.; J. O. Moore, Wilson, N. Y., and Anderson Crowforth, Lockport, N. Y.

Several complaints were made against illegal practising. They were turned over to the Prosecuting Committee, with power. Other very important business pertaining to the profession generally was disposed of. Drs. Stocking and Crowforth presented two interesting papers, which drew forth a lively discussion. The meeting adjourned. Medina will be the next place of meeting, in February, 1901.

ANDERSON CROWFORTH,
Secretary.

The American Public Health Association will meet in Indianapolis, Ind., October 22 to 26th, inclusive. "Animal Diseases and Animal Foods" will be the subject of discussion in the general meeting. In the meetings of the Section on Bacteriology and Chemistry the "Laboratory Work on Tuberculosis" and "Bacteriology of Milk in Its Sanitary Relations" will be fruitful themes of discussion.

